# Weight gain in infancy and metabolic dysfunction-associated steatotic liver disease (MASLD) in a prospective birth cohort of Latino children

**DOI:** 10.1186/s40748-025-00225-8

**Published:** 2025-08-15

**Authors:** Sarah L. Maxwell, Jennifer C. Price, Emily R. Perito, Philip Rosenthal, Janet M. Wojcicki

**Affiliations:** 1 Department of Pediatrics, Division of Pediatric Gastroenterology, Hepatology, and Nutrition, University of California, 550 16th street, 4th Floor, San Francisco, CA 94143 USA; 2 Department of Medicine, Division of Gastroenterology, University of California, San Francisco, USA; 3 Department of Epidemiology and Biostatistics, University of California, San Francisco, USA

**Keywords:** MASLD, Infant weight gain, Early risk factors, Early weight trajectories, Metabolic dysfunction associated steatotic liver disease

## Abstract

**Background:**

Metabolic dysfunction-associated steatotic liver disease (MASLD) is the most common chronic liver disease among U.S. children. Early weight trajectories correlate with obesity, cardiometabolic syndrome, and MASLD in children born small for gestational age.

**Methods:**

We evaluated whether increases in weight-for-age (WAZ) score from 0 to 6 months of life, are associated with MASLD in middle childhood, in two prospective birth cohorts of healthy Latino children (*n* = 136).

**Results:**

After adjusting for confounders, increases in WAZ score from 0 to 6 months of age were associated with a higher risk for MASLD in middle childhood (OR 1.54 95% CI, 1.01–2.36; *p* = 0.046).

**Conclusions:**

In a prospective study of Latino children, increases in WAZ score from 0 to 6 months were associated with increased risk of MASLD in mid-childhood. This could inform early screening and counseling for MASLD.

## Background

Metabolic dysfunction-associated steatotic liver disease (MASLD), the most prevalent chronic liver condition in US children and a leading causes of liver transplantation among adults [1], is increasingly diagnosed in younger children. Recent nationally representative cross-sectional data from the National Health and Nutrition Exam Survey (NHANES) found a 18.5% prevalence of MALSD among adolescents and young adults in the US [2]. Yet the prevalence of MASLD is significantly higher in Latino adolescents (26.9%) compared to either White (9.8%) or Black adolescents (7.9%) [2]. One possible contributor to the higher prevalence of MASLD among Latinos is that a PNPLA3 single nucleotide polymorphism variant (I148M), a lipid regulator associated with hepatic steatosis, steatohepatitis, fibrosis, and cirrhosis [3], is more common among Latinos, with peoples of Mexican and Central American origin having the highest frequency (59–77%) [4]. Although the PNPLA3 variant plays an important role in risk for MASLD, it does not explain all of the increased risk.

Factors such as maternal pre-pregnancy overweight/obesity [5], low and higher birth weights [6], infant weight gain in the first 3 months [7], and body mass index (BMI) at 2 years of age [8] have been found to contribute to pediatric MASLD risk. Breastfeeding protects against MASLD development [9]. Despite identified risk factors, the association between the earliest weight trajectories and increased risk for pediatric MASLD in Latino infants remains unexplored. This study aims to determine if increases in weight for age z score (WAZ) from birth to 6 months is associated with MASLD in middle childhood, in an urban birth cohort of Latino infants.

## Methods

### Study design and subjects

We analyzed data from two longitudinal birth cohorts of Latino children, primarily of Mexican and Central American origins, aimed at identifying early predictors of childhood obesity. Specifically, we sought to assess if the exposure of early weight gain, positive changes in weight for age z scores, from 0 to 6 months is associated with the outcome MALSD in mid-childhood in Latino infants. Women were recruited during pregnancies at the University of California, San Francisco (UCSF) and Chan-Zuckerberg San Francisco General Hospital, the cohorts’ recruitment spanned two periods 2006–2007 (Hispanic Eating and Nutrition [HEN] cohort) and 2011–2013 (Latino, Eating, and Diabetes [LEAD] cohort) [10–12]. Both cohorts included Latina mothers expecting a healthy newborn from non-high-risk pregnancies, excluding insulin dependent diabetic mothers in the HEN cohort, but not in the LEAD cohort. The recruitment protocols have been described previously [10–12]. Contraindications to breastfeeding or Apgar scores < 7 at 5 min of life led to exclusion. The current study was restricted to participants with MASLD laboratory assessments. Annual written consent was obtained. The study was approved by the Institutional Review Board (IRB) at UCSF.

### Measurements

This sub-study, within the broader cohorts, specifically examines maternal, prenatal, and infant risk factors and their correlation with childhood MASLD [8]. Participants in both cohorts had prenatal, birth, six-month, one year, and then annual visits up to 14–15 years of age for HEN and 6–7 years of age for LEAD. Breastfeeding exclusivity, defined using the World Health Organization’s definition [13], and duration of breast feeding were collected in food frequency questionnaires (FFQ) via maternal report at 6 months and 1 year of age. Age at food introduction and amount of sugar sweetened beverage (SSB) consumption, defined as beverages with added sugar including fruit juice and soda, were collected during follow-up visits. Anthropometric measures including length/height and weight were obtained on children using standard digital scales, measuring boards, and stadiometers at each follow-up visit.

Of 298 mother-child pairs in both cohorts, 137 children eligible for this sub-study had serum laboratory tests conducted at either 5–8 years of age (LEAD cohort) or 8–14 years of age (HEN cohort), including: alanine aminotransferase (ALT) and aspartate aminotransaminase (AST) [8]. Children were excluded for serum testing if they were taking medications known to elevate liver function tests or if they had previously diagnosed liver disease. Overweight and obesity were defined based on BMI percentiles using CDC 2000 growth charts with overweight being ≥ 85th percentile for their age and sex and obesity being ≥ 95th percentile for their age and sex [14, 15]. We used the CDC 2000 growth charts, for consistent tracking of growth trajectories from birth through mid-childhood. While the WHO growth standards are often applied to children under 2, the CDC reference was chosen to maintain a uniform standard across all ages in our analysis. Excessive weight gain during pregnancy was defined per American College of Obstetrics and Gynecology (ACOG) guidelines [16]. Rapid weight gain during infancy was defined as a change in weight for age z scores > 0.67 [17, 18]. MASLD was defined if BMI was ≥ 85th percentile for their age and sex and ALT ≥ 95th percentile for age and sex (males ≥ 25.8 IU/L and females ≥ 22.1 IU/L) [15, 19]. These ALT cut-points have sensitivity and specificity for detecting MASLD of 80% and 79% respectively for boys and 92% and 85% respectively for girls [19]. The American Association for the Study of Liver Diseases (AASLD) now recommends these ALT cutoffs for MASLD screening in children [20]. ALT is one of the most used screening methods for pediatric MASLD [21, 22] and has also been shown to determine histologic severity [23, 24].

### Statistical analyses

Descriptive statistical analyses characterized demographics, maternal and infant health characteristics, and infant dietary intake. Normality of the data was assessed using the Shapiro Wilk Test. For continuous variables, a student’s T-test was used for normal distributions and two-sample Wilcoxon rank sum (Mann-Whitney) test was used for non-normal distributions. Chi-squared or Fisher exact tests evaluated differences in categorical variables.

Our primary predictor was change in WAZ score from birth to 6 months of age, calculated by subtracting the WAZ score at birth from the WAZ score at 6 months of age. A WAZ score of 1 unit corresponds to 1 standard deviation from the mean WAZ for the child’s age and sex. A change in WAZ score of 0 to 1 for a six-month-old male corresponds to 1 kg weight increase from 7.9 kg to 8.9 kg. We assessed whether the association between WAZ score and odds of MASLD was approximately linear, we modeled WAZ as a continuous variable and examined the predicted probability of MASLD across the range of WAZ values. Differences in maternal and infant characteristics who received laboratory testing for MASLD were also assessed to assess for potential biases from missing data. Covariates associated with MASLD (*p* < 0.05) in bivariate analysis and covariates known to be associated with MASLD based on prior literature were included in multivariable logistic regression models, including age at MASLD assessment (years), child sex (male versus female), maternal pre-pregnancy BMI (kg/2), and any breastfeeding at 6 months of age (versus none). Infant birth weight or birthweight category were not included in the model due to collinearity with weight change from birth to 6 months. We used STATA 17.0 (StataCorp) for all computations.

## Results

Children in the study were predominantly of Mexican (60%) and Central American (36%) origin; 91% of mothers were primarily Spanish-speaking (Table 1). Median maternal age at delivery was 27 years and median pre-pregnancy BMI was 25.6 with 60% of mothers having overweight/obesity before pregnancy. Gestational hypertension and diabetes mellitus were rare (< 10%) (Table 1).

**Table 1:** Maternal and infant health characteristics and MASLD in middle childhood

	Overall *N* (%) or Median (IQR)	Children without MASLD *N* = 99 (73%)	Children with MASLD *N* = 37 (27%)	*P* values
**Maternal Demographics**				
Maternal country of origin (self-reported)				
*Mexican*	82 (60)	62 (63)	19 (51)	
*Central American*	49 (36)	33 (33)	16 (43)	
*Other Hispanic*	5 (0.03)	4 (4)	2 (5)	0.491
Maternal primary language				
*English*	8 (6)	5 (5)	3 (8)	
*Spanish*	124 (91)	90 (91)	34 (92)	
*Spanish and English*	4 (3)	4 (4)	0 (0)	0.502
Maternal education level (high school diploma or more education)	19 (20)	13 (21)	6 (20)	0.914
Married	128 (94)	92 (93)	36 (97)	0.307
Special supplemental nutrition program for Women, Infants and Children (WIC) participation in pregnancy	128 (94)	92 (93)	36 (97)	0.307
**Maternal Health Characteristics**				
Maternal age, years	27 (23, 31)	27 (23, 30)	26 (22, 32)	0.761
Pre-pregnancy BMI (kg/m2)	25.60 (23.03, 29.43)	25.50 (22.80, 28.40)	27.00 (24.10, 31.10)	0.101
Pre-pregnancy BMI category:				
*BMI < 25 kg/m2 (normal/underweight)*	54 (40)	43 (43)	11 (30)	
*BMI≥25 kg/m2 (overweight/obese)*	82 (60)	56 (57)	26 (70)	0.146
Excess weight gain in pregnancy as per ACOG guidelines	54 (53)	40 (53)	14 (52)	0.895
Gestational hypertension	8 (5.88)	3 (3.00)	2 (5.00)	0.512
Gestational diabetes mellitus	5 (3.67)	6 (6.00)	2 (5.00)	0.885
**Infant Health Characteristics and Infant Dietary Intake**				
Male sex	64 (47)	43 (43)	21 (57)	0.166
Gestational age (weeks)	39.50 (38.64, 40.14)	39.71 (38.86, 40.28)	39.0 (38.43, 40.00)	0.096
Preterm (born at < 37 weeks gestation)	9 (7)	7 (7)	2 (5)	0.728
Birthweight (kg)	3.33 (3.23, 3.42)	3.48 (3.08, 3.71)	3.28 (3.02, 3.45)	0.0828
Birthweight (kg)				
Low birthweight (1,500-2,499 g)	6 (4)	2 (2)	4 (11)	
Normal birthweight (2,500- 3,999 g)	81 (60)	56 (57)	25 (68)	
High birthweight (> = 4,000 g)	49 (36)	41 (41)	8 (22)	0.017
Small for gestational age	11 (8)	7 (7)	4 (11)	0.477
WAZ score at birth	-0.06 (-0.64, 0.40)	0.06 (-0.64, 0.57)	-0.14 (-0.63, 0.08)	0.106
WAZ score at 6 months	0.27 (-0.37, 0.91)	0.25 (-0.46, 0.84)	0.53 (-0.26, 1.25)	0.211
Rapid weight gain from birth to 6 months (*≥* 0.67 SD change in WAZ)	54 (43)	37 (40)	17 (50)	0.325
Change in WAZ score from birth to 6 months	0.51 (-0.32, 1.21)	0.42 (-0.50, 1.11)	0.69 (0.01, 1.68)	0.019
Any breastfeeding at 4–6 weeks of age	122 (91)	88 (91)	34 (92)	0.832
Exclusive breastfeeding at 4–6 weeks of age	44 (33)	32 (33)	12 (33)	0.970
Any breastfeeding at 6 months of age	84 (65)	64 (69)	20 (57)	0.215
Exclusive breastfeeding at 6 months of age	26 (21)	20 (23)	6 (18)	0.588
Any breastfeeding at 12 months of age	59 (47)	45 (49)	14 (41)	0.410
Any sugary beverage intake at 6 months	63 (53)	46 (53)	17 (52)	0.894

Among infants, 93% were born full term and 59% were born with normal birthweights. Most infants were breastfed, with 33% of mothers breastfeeding exclusively at 4–6 weeks of age and 21% exclusively breastfeeding at 6 months of age (Table 1).

### MASLD prevalence

**Fig. 1:**
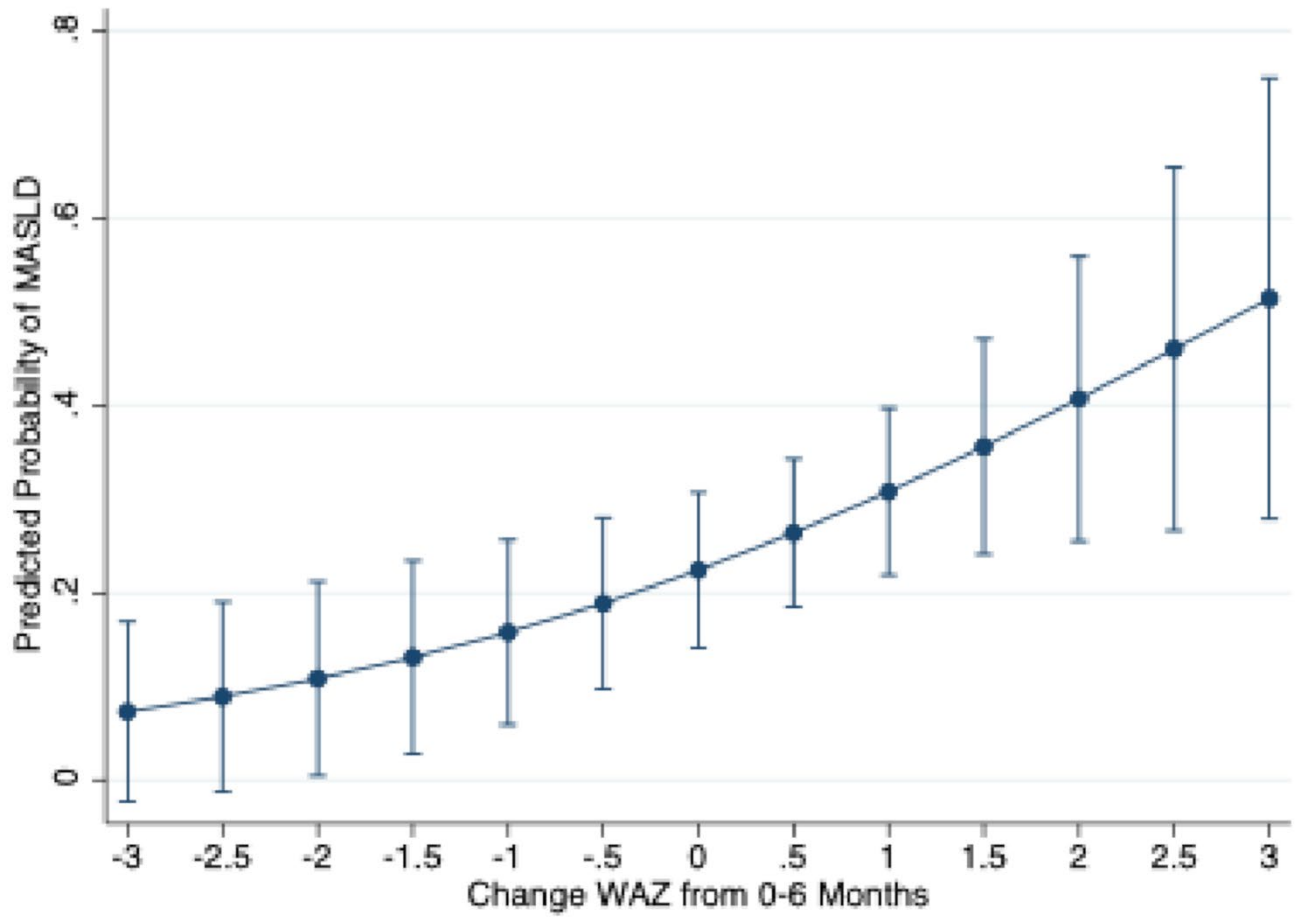
Adjusted Predicted Probability of MASLD by Change in Weight for Age Z Scores with 95% Confidence Intervals

The prevalence of MASLD in mid-childhood (5–12 years of age) among the 136 children was 27%. There were no statistically significant differences in maternal ethnicity (Latina versus non-Latina), language (Spanish versus English/Bilingual), or WIC participation (yes or no) in children who had undergone labs for MASLD assessment, compared to those without labs for MASLD assessment. Children with MASLD had slightly lower median birthweights, 3.28 kg (IQR: 3.02, 3.45) compared to those without MASLD, 3.48 kg (IQR: 3.08, 3.71), but the difference was not statistically significant. Children with MASLD had a slightly lower median WAZ scores at birth − 0.14 (IQR: -0.63, 0.08) compared to those without MASLD: 0.06 (IQR: -0.64, 0.57), though this was not statistically significant *p* = 0.106. By 6 months of age, the children with MASLD had higher median WAZ scores 0.53 (IQR: -0.26, 1.25) compared to those without MASLD: 0.25 (IQR: 0.46–0.84), though this also was not statistically significant (*p* = 0.211). The children with MASLD showed a significantly higher median change in WAZ from birth to 6 months compared to those without MASLD: 0.69 (IQR: 0.01, 1.68) vs. 0.42 (IQR: -0.50, 1.11) respectively, (*p* = 0.019). Approximately, 78% of the children who developed MASLD had a positive change in WAZ score from 0 to 6 months vs. 61% of those who did not develop MASLD, *p* = 0.05. In our cohort, the predicted probability of MASLD increased with higher change in WAZ scores from 0 to 6 months of age (Fig. 1); this demonstrates that, as change in WAZ score increases in this cohort, the likelihood of having MASLD also increases steadily. Neither exclusive nor any breastfeeding before 6 months of age were associated with MASLD in this cohort, though rates of exclusive breastfeeding were low (Table 1).

### Predictors of MASLD in the multivariate model

In multivariable logistic regression, every one unit increase in change in WAZ score from birth to 6 months was associated with 54% increased odds of MASLD (OR 1.54 95% CI: 1.01, 2.36; *p* = 0.046), after adjusting for age at MASLD assessment (years), child sex, maternal pre-pregnancy BMI (kg/2), and any breastfeeding at 6 months of age. Female sex was also associated with a 60% decreased odds of MASLD compared with male sex (OR 0.40 95% CI: 0.16, 0.99, *p* = 0.049; Table 2). Child’s age at MASLD assessment, maternal pre-pregnancy BMI, and any breastfeeding at 6 months were not statistically significant in this model (Table 2).

**Table 2:** Multivariable model of predictors for MASLD in middle childhood

Variable	Unadjusted OR (95% CI)	*P* value	Adjusted OR (95% CI)	*P* value
Child age at assessment, years	1.16 (95% CI 0.99,1.37)	0.050	1.13 (95% CI 0.95, 1.35)	0.177
Sex (reference = male)	0.58 (95% CI 0.27,1.25)	0.168	0.40 (95% CI 0.16, 0.99)	0.049
Any breastfeeding at 6 months (reference = none)	0.60 (95% CI 0.27,1.34)	0.217	0.62 (95% CI 0.24,1.57)	0.313
Maternal pre-pregnancy BMI (kg/m2)	1.06 (95% CI 0.99,1.14)	0.082	1.06 (95% CI 0.99, 1.14)	0.109
Change in WAZ score from birth to 6 months	1.54 (95% CI 1.06, 2.23)	0.022	1.54 (95% CI 1.01, 2.26)	0.046

## Discussion

In this cohort of 5–12 year-old Latino children born in San Francisco, we found a MASLD prevalence of 27.0%, which is notably comparable with the 26.9% prevalence =from the NHANES cohort in an older age group of children, 12-17 year old Latino adolescents [2]. As MASLD prevalence is known to increase with age, we anticipate that this high-risk group will have a higher prevalence of MASLD when they reach adolescence. We had previously found that higher BMI-z scores at 2 years of age were associated with increased risk for MASLD in mid-childhood [8]. In this paper we sought to explore how the earliest weight gain from 0 to 6 months influences pediatric MASLD risk. We found that greater changes in WAZ score from birth to 6 months of age was associated with an increased risk for MASLD in middle childhood, specifically for every 1 unit increase in WAZ score, the odds of MASLD increases by 54%. This novel finding persisted after adjusting for age of MASLD assessment, infant sex, maternal pre-pregnancy BMI (kg/m2), and breastfeeding status at 6 months of age. This is the first study to link early infant weight gain in healthy Latino infants with MASLD in middle childhood, underscoring the potential importance of even very early-life weight gain in influencing later liver disease risk. The overall prevalence of exclusive breastfeeding was low in our cohort (21%) and while we did not find any association between exclusive BF and MASLD, other studies have found that exclusive BF is protective against rapid infant weight gain [25, 26], childhood obesity [27], MASLD [9, 28], and fibrosis [9].

Our findings are consistent with prior literature that demonstrates that early weight gain is associated with childhood obesity [17, 18, 29–31] as well as with MASLD in infants born small for gestational age [32]. A study in the Netherlands found weight gain in the first 3 months of life was associated with a higher risk for MASLD in early adulthood regardless of birth weight [7]. Similar to our findings, longitudinal studies of Asian children in Singapore have found that BMI accelerations in infancy and early childhood (0–2 years) increase risk for obesity, abdominal fat, liver fat, and other cardiometabolic conditions [33, 34]. Another longitudinal population-based study identifying cardiovascular risk factors for MASLD in Finland found that those who developed MASLD had lower birthweights than those who did not and relatedly that being born small for gestational age was also associated with adult MASLD [35]. These findings are consistent with research on “rapid catch-up growth” in infancy being associated with later development of cardiometabolic syndrome [35, 36], including MASLD [7]. This further suggests that risk for MASLD may start early and children at higher risk for MASLD and other obesity related comorbidities may be able to be identified for interventions in infancy.

Limitations of this study include the absence of imaging or liver biopsy for MASLD confirmation. It was not feasible in this cohort study of healthy children to perform biopsies or imaging on all overweight patients with elevated transaminases. Our definition of MASLD is based on a study which found that these laboratory cutoffs had a 80% sensitivity for MASLD in boys and 92% in girls and a 79% specificity for boys and 85% for girls [19], which likely leads to an underestimation of MASLD prevalence. The AASLD’s new practice statement also recommends using these ALT values for MASLD screening in children [20]. Although the NASPGHAN recommendation of using ALT > 2x upper limit of normal prioritizes specificity [37], it may miss some children at high risk. Our approach prioritizes early identification in a high-risk population. Although ALT can be elevated in other conditions, in this Latino cohort of children with a high prevalence of obesity at high risk for the PNPLA3 I148M allele, elevated ALT values were most likely due to MASLD. The sample size (*n* = 136) is also relatively small, which may explain why we see some trends in maternal pre-pregnancy BMI (higher risk with higher BMI) and breastfeeding (protective against MASLD) that are in the expected direction, but not reaching statistical significance. The association between change in WAZ score and MASLD had an OR of 1.54 with a 95% confidence interval ranging from 1.01, 2.36, indicating that our estimated value is substantial, however, the confidence interval does go down as low a 1% interval, but our best estimate is an approximate 54% increase. Lastly, timing of complementary feeding and comprehensive dietary intake in the first 6 months of life was not assessed in this cohort.

Strengths of the study include the longitudinal design with well-characterized maternal and infant risk factors and inclusion of low-income Latino children who are exclusively of Mexican and Central American origins and at high risk for MASLD.

## Conclusions

Greater WAZ scores increases in Latino infants from birth to 6 months of age are linked to heightened risk for MASLD in middle childhood. Our findings imply that pediatricians should identify those at risk for early weight gain and MASLD, monitor their growth closely, encourage exclusive breastfeeding until 6 months of age, and provide anticipatory guidance on healthy feeding practices in early childhood including delaying complementary feeding until 6 months of age. Additional, larger studies are needed to better understand the pathophysiology between higher weight gain in the first 6 months of life, dietary intake and the future risk of MASLD in high-risk children.

## Data Availability

The datasets used and/or analysed during the current study are available from the corresponding author on reasonable request.
